# Auricular Capillary Hemangioma: A Rare Case Report

**DOI:** 10.1002/ccr3.71542

**Published:** 2025-12-01

**Authors:** Mahboobe Asadi, Amirhossein Ehsani, Zhaleh Mohsenifar

**Affiliations:** ^1^ Department of Otolaryngology and Head and Neck Surgery Shahid Beheshti University of Medical Sciences Tehran Iran; ^2^ Department of Pathology Shahid Beheshti University of Medical Sciences Tehran Iran

**Keywords:** capillary hemangioma, case report, external ear, vascular neoplasms

## Abstract

Auricular capillary hemangioma in an adult patient is a rare entity that should be considered in the differential diagnosis of vascular lesions of the external ear. Complete surgical excision has a low recurrence rate with excellent cosmetic outcomes.

## 1 Introduction

Capillary hemangiomas are benign vascular tumors characterized by proliferating capillary‐sized vessels. They usually present in infancy and involute spontaneously. Adult‐onset capillary hemangiomas of the auricle are rare and can pose diagnostic challenges due to their similarity to other vascular or inflammatory lesions [1, 2]. Histopathological examination remains the gold standard for diagnosis. Due to the external ear's anatomical and cosmetic significance, prompt diagnosis and treatment are necessary [3].

## 2 Case History

**FIGURE 1:**
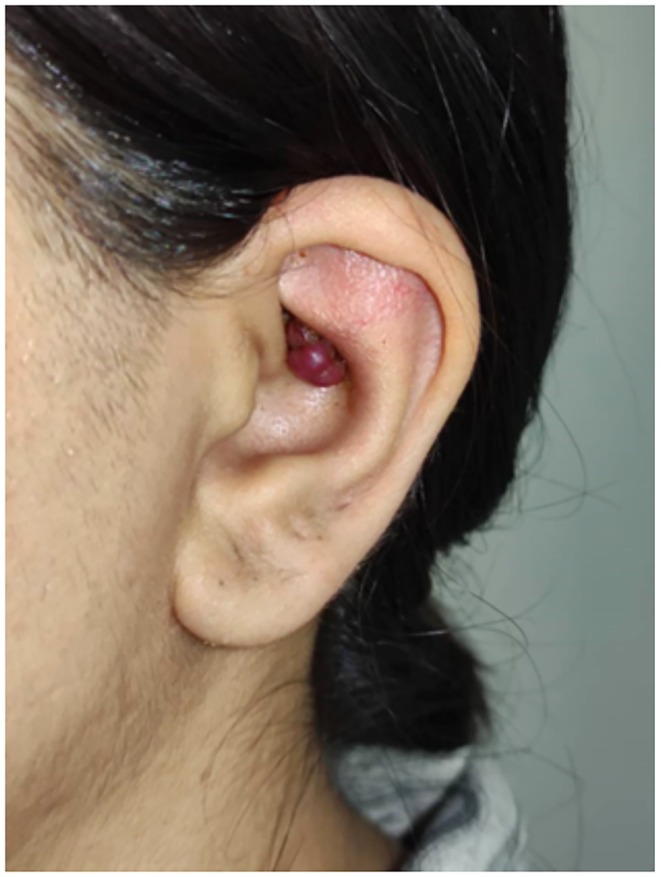
Capillary hemangioma of the left auricle.

A 32‐year‐old woman presented with a 6‐month history of a gradually enlarging, reddish nodular lesion on the left auricle. She also reported episodes of bleeding following minor trauma. There was no history of trauma or systemic disease. Physical examination revealed a 1.2 cm, lobulated, erythematous lesion on the left cymba concha (Figure 1). The external auditory canal and the tympanic membrane were normal.

## 3 Differential Diagnosis, Investigations, and Treatment

Differential diagnoses of a solitary polypoid red lesion of the pinna include cholesteatoma, carcinoma, pyogenic granuloma, and vascular tumors. The patient underwent surgical excision under local anesthesia. The lesion was excised completely with a narrow margin of normal tissue. Hemostasis was achieved using bipolar cautery. The defect was closed primarily with 5‐0 nylon sutures.

## 4 Conclusion and Results (Outcome and Follow‐Up)

**FIGURE 2:**
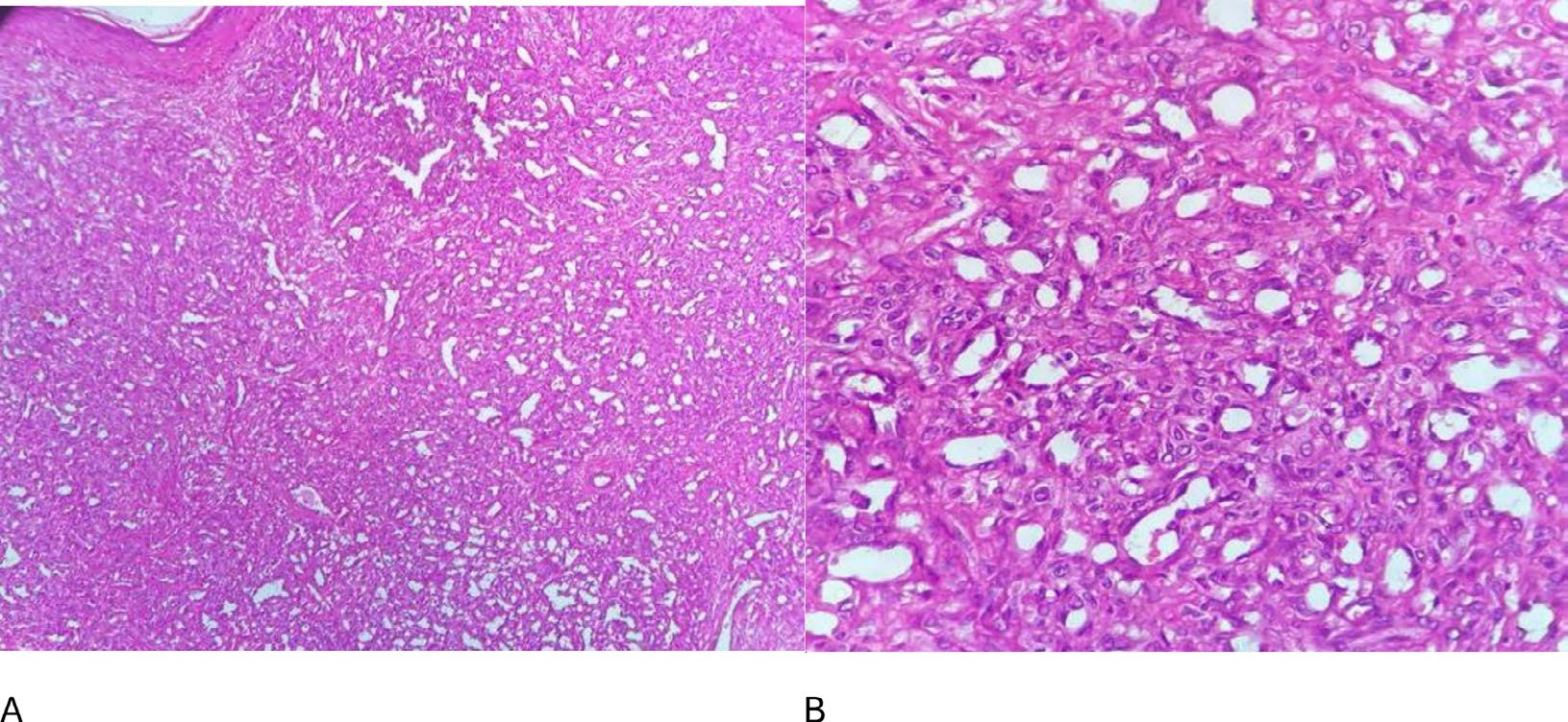
A: Low magnification shows a hematogenous vascular tumor without a capsule, covered by squamous epithelium. B: High magnification shows vessels having a single layer of flat endothelial cells without mitosis or atypia.

**FIGURE 3:**
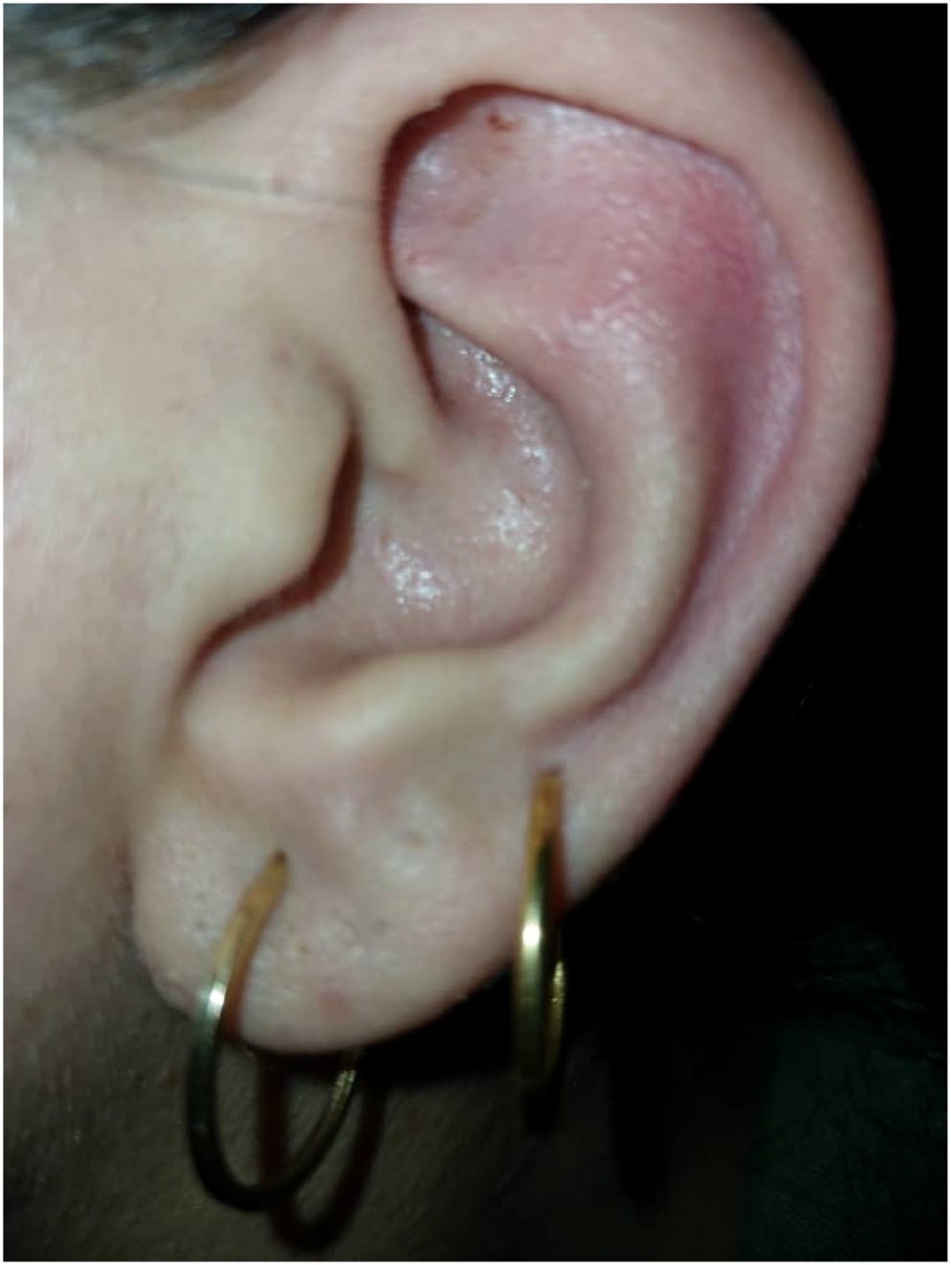
Three months postoperative view.

Histopathological analysis showed densely packed capillaries lined by a single layer of flattened endothelial cells without atypia confirming the diagnosis of capillary hemangioma (Figure 2). The patient's postoperative course was uneventful. At 3‐month follow‐up, there was no evidence of recurrence, and the patient was satisfied with the cosmetic outcome (Figure 3).

## 5 Discussion and Literature Review

Capillary hemangiomas are predominantly pediatric lesions with peak incidence in infancy. Adult cases are rare and less characterized. Hemangiomas of the external ear (the auricle and external auditory canal) and occasional extension into the tympanic membrane or middle ear are rare in adults. Based on an English‐language literature review, approximately 30 adult cases of temporal bone hemangioma (capillary, cavernous, and mixed subtypes) have been reported up to 2025 [4]. Besides, hemangiomas of the auricle are extremely rare with only two cases of arteriovenous hemangioma reported in the ear lobule [5] To the best of our knowledge, ours is the first reported case of a capillary hemangioma in the pinna.

Although, infantile hemangiomas have a distinct proliferative and involution phase linked to angiogenic factors such as VEGF, the etiology of adult‐onset hemangioma is still unknown. However, the pathogenesis of adult capillary hemangiomas is not fully understood; localized vascular proliferation triggered by minor trauma, hormonal changes and hypoxia has been discussed [1, 6]. Differential diagnoses include cholesteatoma, carcinoma, pyogenic granuloma, and other vascular tumors. Temporal CT scan is commonly helpful in the primary diagnostic approach that assists in determining the extent of the lesion, bony erosion, and serves as a guide for surgical approach. Besides, angiography may assist in recognizing feeding blood vessels. However, histopathological examination remains the gold standard for definitive diagnosis, showing clusters of capillary vessels lined by benign endothelial cells without atypia or mitoses [7]. Unlike external auditory canal and middle ear hemangiomas that present with hearing problems such as aural fullness, hearing loss and tinnitus, in auricular hemangioma a vascular lesion maybe the only manifestation [8].

Surgical excision is the treatment of choice for the adult hemangioma occurring in the ear, especially when the tumor causes cosmetic problems [5].

Moreover, the literature review revealed that in EAC hemangioma endaural, transcanal, or retroarticular removal is the common effective approach [8].

The pinna has a unique three‐dimensional structure composed of cartilage and skin. In any surgical procedure involving the auricle, the convex and concave structures of the auricle need to be preserved [9]. In this case, the mass had small dimensions, so the defect was closed with primary closure. In summary, we presented an anatomically rare presentation of capillary hemangioma in which cosmetic considerations were important.

## 6 Conclusion

Capillary hemangioma arising from the auricle is rare. Although ear hemangiomas are asymptomatic in most cases, surgical excision is the treatment of choice.

## Author Contributions

**Mahboobe Asadi:** conceptualization, investigation, writing – original draft, writing – review and editing. **Amirhossein Ehsani:** data curation, validation, writing – original draft, writing – review and editing. **Zhaleh Mohsenifar:** investigation, writing – original draft, writing – review and editing.

## Funding

The authors have nothing to report.

## Ethics Statement

Ethical approval for this study was approved by the Ethical Committee of Shahid Beheshti University of Medical Sciences, Tehran, Iran. The present study complies with ethical and research standards involving humans. This article does not contain any studies involving animals performed by any of the authors.

## Consent

Written informed consent was obtained from the patient for publication of this case report and the accompanying images. A copy of the written consent is available for review by the editor‐in‐chief of this journal.

## Conflicts of Interest

The authors declare no conflicts of interest.

## Data Availability

Data in the current study are available from the corresponding author on reasonable request.
